# Estimating reach of social impact products: A model to standardize the calculation of product reach in data-scarce settings

**DOI:** 10.29392/joghr.3.e2019029

**Published:** 2019-06-01

**Authors:** Svenja M Jungjohann, Emily Carnahan, Peiman Milani, Cyril Engmann

**Affiliations:** 1 Global Alliance for Improved Nutrition, Geneva, Switzerland; 2 PATH, Seattle, WA, USA; 3 Sight and Life, Basel, Switzerland

## Abstract

**Background:**

Social impact interventions often involve the introduction of a product intended to create positive impact. Program decision makers need data to routinely review product delivery as well as predict potential outcomes and impact to optimize intervention plans and allocate resources effectively. We propose a novel model to support data-driven decision-making in data and budget-constrained settings and use of routine monitoring to ensure progress towards program outcomes and impact.

**Methods:**

We present a complete model to estimate product reach of durable and fast-moving consumer products, which includes required inputs, potential data sources, formulas, trade-offs, and assumptions.

**Results:**

We illustrate the use of the model by applying it to the case study of fortified rice introduction in Brazil and estimate that the intervention, which aimed to improve nutrition status and health outcomes reached 2.4 million consumers.

**Conclusions:**

The model can cover a broad range of social-purpose interventions that involve the introduction or scale-up of various types of consumer products. It provides a relatively simple, comprehensive, flexible, and usable framework to estimate product reach, an indicator that can be an input into impact estimates or, in many scenarios, the actual endpoint of the intervention.

The Sustainable Development Goals (SDGs) propel governments, donors and implementing agencies to invest in evidence-based, efficacious social impact interventions to improve the situation for people (*1*, *2*). To attain the SDGs a range of these programs or interventions need to be implemented effectively to achieve programmatic and policy goals. How inputs and activities of each intervention are expected to generate value and positive impact in the target population (those with the greatest potential to benefit) is outlined in the intervention’s impact pathway. Delivery bottlenecks often hinder and, if not addressed, prevent interventions from achieving their intended effects. Quantifying the intervention’s value creation process is important to identify and address delivery bottlenecks and to ensure that efficacious intervention models can effectively attain the desired results in the applied context (*3*).

Agencies implementing and managing such interventions therefore require delivery and utilization data to make informed decisions to optimize plans and reallocate resources effectively. Resources dedicated to this cause are limited for interventions in developing countries, particularly for delivery research and routine monitoring (*3*, *4*).

Program sponsors, such as government policymakers and funding agencies, need to understand if the interventions they invest in are generating the desired strategic outcomes and impact in the population (value for money). Measuring population outcomes to understand who is benefiting from the intervention requires investments into surveys, which compete for program funds with other intervention activities. Survey data is therefore not frequently available and overemphasis on measuring at the population level can divert attention of program managers and program funds from routine monitoring and effective management of intervention delivery.

Social impact interventions often involve the introduction of a product intended to create positive impact for a large number of people – be it health, social, environmental, or economic in nature (*5*). Products have been introduced in low, middle and high-income settings worldwide and can be grouped into two types of consumer goods:

- Durable consumer goods: products that yield utility over time rather than being completely consumed in one use. These may be one-time purchases or goods with long periods between successive purchases. Examples include latrines, LED/solar lamps, bed nets, irrigation pumps, and menstrual cups.
- Fast-moving consumer goods: products that are immediately consumed in one use or have a short lifespan, usually no more than a few months. These typically represent repeated purchases over a period of time. Examples include condoms, fortified foods or supplements, contraceptive pills, and oral rehydration solutions.

Program decision makers need monitoring data to routinely review product delivery as well as predict potential outcomes and impact to make timely adjustments to intervention plans and allocate resources effectively. Facing scarcity of robust data and direct beneficiary registration in low-resource settings, organizations from different fields have turned to modeling approaches using data that are tracked and can be obtained from one or more nodes in the product supply chain to estimate product reach. In the field of vaccines, for example, vaccine coverage for a specific antigen can be estimated based on the number of immunizations purchased or distributed within a region (*6*, *7*). To estimate intake of food products, WHO and GAIN have proposed food consumption methodologies that model data from twenty-four hour recall, food frequency questionnaires, food balance sheets, or household consumption and expenditure surveys (*8*, *9*). These challenges in estimating product reach are not restricted to commercial distribution scenarios, they also apply when the primary distribution channel is the health system or another safety net channel in a low-income country.

A standardized model that harmonizes the use of available data sources to report on implementation progress and expected product reach among the population would be a valuable tool for decision-makers funding or implementing social impact interventions. A product can only achieve social impact in the target population if it is used by them. The model presented here illustrates how available data from nodes along the product supply chain from production to consumption can be triangulated to estimate the number of individuals potentially using and benefitting from the product. The model distinguishes two main ways to estimate product reach of a defined target population: one using product coverage data directly measured in the target population, and the other using data on individual utilization patterns coupled with supply volumes of a fast-moving or durable product. This model can be equally applied for both types of products – durable and fast-moving – and across fields (immunization, nutrition, reproductive health, etc.).

This paper describes the model in detail, illustrates its application for a public health intervention with the case of fortified rice in Brazil and discusses its applicability, strengths, and limitations. The model draws on multiple data sources across the supply chain and can be tailored to the available data sources, drawing on explicit assumptions to fill in the gaps. The model was designed to be utilized within the real-world, funding-constrained, and data-scarce environment that most social impact initiatives operate in.

## The model for estimating product reach

**Figure 1:**
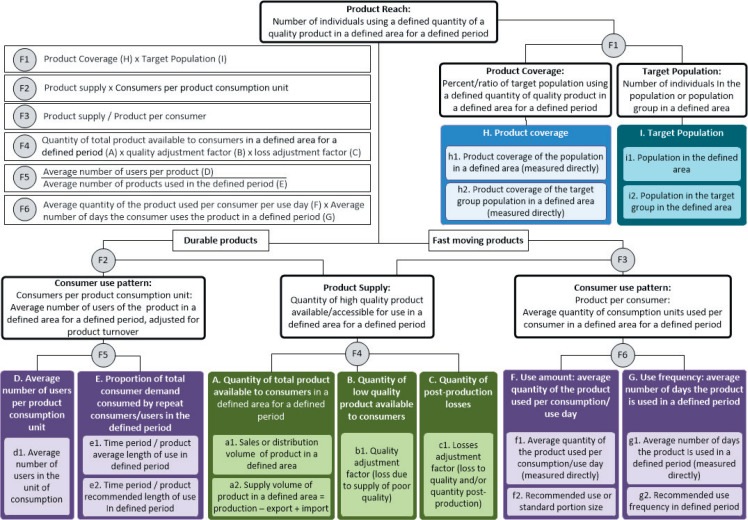
A model to estimate product reach for durable and fast moving products.

**Figure 1** outlines the model to estimate **product reach** of social impact products in two main ways:

1. by multiplying (F1) the **product coverage** or ratio of a given population that use a defined quantity of the product (H) by the number of individuals in that **target population** (I)
2. by combining data on **product supply** (production, sales/distribution) and **consumer use patterns**, which are estimated differently for durable (F2) and fast-moving consumer goods (F3).

The boxes in the figure represent key data elements that are inputs to the model (denoted with A-I) as well as their measure(s) used to quantify each data element (denoted with a-i). Multiple boxes indicate there are different measurement options available that can be used as proxies to calculate the data element. We next explore each data element in the model and consider measurement options, data sources, and assumptions, which are detailed in **Table 1**.

**Table 1:** Data sources, considerations and assumptions for each measure within the product reach model

MODEL ELEMENT	MEASURE	DATA SOURCE	CONSIDERATIONS & ASSUMPTIONS
Product supply					
A. Quantity of total product available to consumers in a defined area for a defined period	a1. Sales or distribution volume of product in a defined area	- Distribution data	- Sales data can be localized to the point of sale which can provide more detailed estimates of where the product is ending up with the consumer.
		- Point-of-sale data	- A ssume that all sales or distribution that occur in the target area translate into use of the product in that area. One could adjust this assumption by multiplying by a factor that accounts for product supply leakage outside of the defined area.
	a2. S upply volume of product in a defined area=production–export+import	- Manufacturer data (production and inputs)	- Production, import, and export data is often less localized than sales data.
		- Distribution and export data	- Need to account for illegal imports or contraband that may enter the market in the defined area.
		- Import data	- In the absence of import and/or export data, assume that all production that occurs within the target area remains as supply within the target area. (This makes more sense when the target area is at a national or regional level, not smaller.)
B. Quantity of low quality product available to consumers	b1. Quality adjustment factor (loss due to supply of poor quality) *This is a number (0 to 1) where 1 represents that 100% of the product is high quality.*	- External monitoring of product quality (production site inspections) - Import monitoring of product quality - Market or commercial monitoring of product quality	- T o maximize product reach, assume that there are no product quality issues in production and product adheres to standard (quality adjustment factor = 1).
C. Quantity of post production losses	c1. Losses adjustment factor (loss to quality and/or quantity post-production) *This is a number (0 to 1) where 1 represents that 100% of the product is available and high quality.*	- Manufacturer data on supply chain losses - Distribution data on losses - Point of sale data on losses - Market or commercial monitoring of product quality - Secondary data on other similar products	- Data is unavailable, use data from a similar product in the market or from the same product in a different market. - To maximize product reach, assume that there are no product losses post-production (losses adjustment factor = 1).
Consumer use pattern:					
D. A verage number of d1. A verage number of users in the unit of users per product consumption	d1. Average number of users in the unit of consumption	- Product specifications - Consumer survey - Secondary data on other similar products	- If the product is intended for a single individual’s use, =1 - If the product is intended for household use, use the average number of individuals in a household in the defined area. - If the product is intended for community use, use the average community size in the defined area.
E. Proportion of total consumer demand consumed by repeat consumers/users in the defined period (Durable products)	e1. Time period / product average length of use in defined period e2. Time period / product recommended length of use in defined period	- Time period of calculation defined by project team - Average length of use from market or consumer survey (direct data collection) - Time period of calculation defined by project team - Recommended length of use from product specifications or industry standards	- The time period of estimation is defined by the project team. It may be informed by the time period of the available data or the time period of the project. - The time period of estimation is defined by the project team. It may be informed by the time period of the available data or the time period of the project.
F. Use amount: average quantity of the product used per consumption/use day (Fast-moving products	f1. Average quantity of the product used per consumption/use day (measured directly)	- Market or Consumer survey (direct data collection) - Secondary data on other similar products	- If data is unavailable, use data from a similar product (eg, average consumption of the unfortified version of the food) - If data is unavailable for the specific target population, use for the general population and assume consumption patterns are similar.
	f2. Recommended use or standard portion size	- Producer or project recommended use - Industry standards	- For example, for fortified foods standard portion size may be the amount in a single serving.
G. Use frequency: average number of days the product is used in a defined period (Fast-moving products	g1. Average number of days the product is used in a defined period (measured directly)	- Market or Consumer survey (direct data collection) - Secondary data on other similar products	- More detailed when disaggregated by subgroup, but more complex and potentially more difficult to explain. - Should take into account repeat vs. dropout vs. new consumers. - The default will depend on whether the project prefers a conservative or optimistic estimate of product reach.
	g2. Recommended use frequency in a defined period	- Producer or project recommended use frequency - Industry standards	- Less detailed than disaggregated consumption frequencies by consumer subgroups (e2), but more simplistic and easier to explain.
Product coverage and target population					
H. Product coverage	h1. Product coverage of the total population in a defined area (measured directly) h2. Product coverage of the target group population in a defined area (measured directly)	- Coverage survey - Coverage survey	
I. Target population	i1. Total population in a defined area i2. Target group population in a defined area	- UN estimates - Census data - Project data - UN estimates - Census data - Project data	

## Product reach: number of individuals using quality product

Product reach refers to the estimated number of individuals in a defined area using a defined quantity of product for a defined period.

Depending on the data sources used and the assumptions included in the model, the definition of reach varies. While reach based on the indirect measure of product production or sales/distribution data will estimate the number of individuals for whom a defined amount of the product is available or accessible, respectively, and who can potentially use the product, estimating the number of people that are actually using a defined quantity of the product will need to be based on direct use or consumption data from beneficiary registers or surveys.

Product reach can be estimated for the population or a specific population subgroup in a defined area. For example, if the product channel is the national market without targeted marketing, then the entire population of that country is the target population in the defined area. Assuming equal consumption across population subgroups, the number of individuals reached of a sub group can be estimated by multiplying the total reach by the proportion of the subgroup in the target population using the product. If the product is targeted to a specific population subgroup, the product reach of that subgroup is based on the product supply and the average individual product use of that subgroup.

## Product coverage: ratio of target population using the product

*Product coverage* and product reach are similar measures, but coverage is presented as a percentage of a specific population and reach is an absolute number of individuals. Product coverage can be translated into reach (and vis versa) by multiplying the coverage of a product or service for a given population (H) by the number of individuals in that target population (G). This can either be estimated for the entire population in the defined area (h1, i1) or for a specific target group (eg, children under 5 years of age) that represents a subset of the population in the defined area (h2, i2). Just as for reach, coverage has proxy indicators that have different definitions depending on the data it is based on (availability, accessibility, use, and use of desired quantity to achieve impact (*10*, *11*)).

## Product supply: quantity of high quality product available/accessible for use

In the absence of robust individual-level data, often the most attainable data to calculate product reach is from product supply data available from different levels of the product supply chain (production, dispatch, sales, and/or distribution). Most projects or interventions interested in estimating product reach have a relationship with manufacturers, sellers, or distributors of the product from which regular supply-side information can be obtained. A precursor to estimating product supply is to determine the project’s geographic area and to only consider supply data that refers to the defined area (a neighborhood, city, province, country, or region) and target population. Three key supply-side inputs are quantity of total product available/accessible to consumers (A), quantity of product supplied with quality below product standards or specification requirements (eg, contraceptive pills that do not contain the required level of hormones) (B), and volumes lost post-production in the supply chain (C). Then the total supply (A) less the volumes lost to quality (B) or other post-production issues (C) represents the supply that ultimately matters: the amount of the product that is actually available or accessible to the targeted population group at the desired level of product quality (F4).

The quantity of total product available/accessible can be measured in one of two ways:

a1. The dispatch, sales or distribution volume of the product in the defined area, which can be measured directly, or

a2. The total product supply volume in the defined area that is calculated as the amount of the product produced in the defined area less the amount of product exported outside the defined area plus the amount of product imported into the defined area.

Measure a2 is based on broad availability of the product to consumers based on production, and measure a1 is based on a more direct measure of consumer accessibility to the product through retail outlets or distribution sites. In cases where complete sales or distribution data is available and can be localized to the point of sale/distribution (for example, a specific store or a wholesaler supplying retail outlets in a specific area), measure a1 is preferable since it is a better proxy for consumer accessibility. This would be useful for interventions that anticipate significant differences in product reach, or number of individuals using the product, across geographic areas, be they communities, regions, or countries. Alternatively, if localized data is not integral to the project’s target population or sales data is not available from all retailers, supply data including local production and accounting for imports and exports (a2) may be used. The production amount should only include the amount of product that is dispatched in the defined area. In some markets, one may need to account for illegal contraband products that are entering the market but would not be captured in official data on imports (*12*).

The product supply is an estimate for the quantity of the product that is being used by consumers in a defined area for a defined time period. Thus, the model assumes that all sales or distribution that occur in the defined area (a1) or all product volume that is available in the defined area (a2) directly translate into product use by consumers in that area. These measures should be adjusted to exclude any leakage to consumers outside of the defined area (this leakage can be thought of as exports in a2). Or, if there are market imperfections creating barriers to consumer purchase or excess product supply that is not purchased, the product supply quantity should be adjusted to reflect only the fraction of product that is purchased and subsequently used by consumers during the defined time period.

While box A already excludes any low-quality product that was discarded by the manufacturer through existing quality control processes, box B further adjusts the product supply for any low-quality product that is made available. The quality adjustment factor (b1) should be a number between 0 and 1, where 1 represents that 100% of the product is high quality. This is an especially relevant adjustment in markets with poor regulation or monitoring, or for products where specifications cannot be easily verified and thereby controlled by authorities, retailers, or consumers.

Supply-side losses that occur after production are referred to in the model as post-production losses (C). These represent leakages during transport to or storage at retail outlets or distribution sites, to the point where the product does not reach the target population or no longer meets specifications when it does. For example, a vaccine may be produced according to specifications but if it is not refrigerated properly its efficacy may be decreased by the time it reaches the beneficiary. Post-production losses would also include product shipments lost or destroyed due to expiry or contamination or supplied outside the targeted population group. Post-production losses are represented as an adjustment factor (c1) between 0 and 1, where 1 represents that 100% of the product is available and high quality (i.e. no product is lost post-production). The quantity of total product available to target population (A) is then multiplied by the quality adjustment factor (B) and the post-production losses adjustment factor (C) to give the quantity of high quality product available for use in the defined area for a defined period (F4).

The product supply calculation is the same for durable goods and fast-moving consumer goods. The razor-and-blade business model combines a one-time purchase of a durable good (the “razor”) and multiple purchases over time of a fast-moving good (the “blade”) for replenishment or maintenance (*13*). Point-of-use water filters are an example of a razor-and-blade good since the water dispenser is a one-time purchase (“razor”) and it requires purchasing filter refills on a more regular basis (the “blades”). For these razor-and-blade goods, product supply should be calculated separately for the razor and for the blade. If only data for either the razor or blade is available, then that component part can be used to estimate the reach of the combined product.

## Consumer use patterns

For durable goods, product supply data closely ties to the number of target population since these are often one-time or long-term purchases. For example, we can assume that each sale of a menstrual cup equates to one female user. Similarly, we can assume that if a household purchases a latrine, then all individuals in the household are using the latrine to some degree, which equates to the number of individuals in the household. For the estimation of reach with durable goods (F2), the average number of users per product (D) is multiplied by the average number of products used in the product reach estimation time frame (E) to adjust for product turnover. The menstrual cup’s unit of use is the individual and the average number of users in that unit is one. The latrine’s unit of use may be the household and on average there may be five users in a household in the defined area. Where more disaggregated data are available, they can be included as inputs; for example, to say that 30% of households in the defined area have an average of 4 latrine users, 50% of households have an average of 6 latrine users, and the remaining 20% of households have an average of 9 latrine users.

When the product’s average length of use is longer than the defined period, product turnover is not an issue and E can be equal to 1 (i.e. each individual is using 1 product during the defined period). In other cases, it can be measured in two main ways:

e1. The time period divided by the product’s average length of use, or

e2. The time period divided by the product’s recommended length of use.

For example, if a menstrual cup is used for an average of 1 year, but product reach is estimated over a 5-year period, an individual will use an average of 5 menstrual cups in that defined period. This durable good turnover needs to be accounted for in the model, so the model includes the average number of products used in the defined time period (E). The product’s average length of use (e1) is a preferred model input since it is based on consumer data and therefore a more accurate reflection of how a product is actually used than the recommended length of use (e2).

In contrast to durable goods, many fast-moving consumer goods must be used repeatedly to produce the desired impact. For contraceptive pills to fully deliver their benefits, they must be consumed in adequate quantities on a regular basis. Sales may not be an adequate measure of reach, as it doesn’t correspond to actual use of the product. Therefore, we need to know the amount of product the consumer uses, which is a combination of the used amount and frequency.

For the estimation of reach with fast-moving consumer goods (F3), the product supply (F4) is divided by the product use per consumer (F6). The latter is calculated by multiplying the average quantity of the product that is used per consumer per use day (F) by the average number of days the consumers uses the product in a defined period (G). The default time period we have included in the model is use per day, but this can be adjusted depending on the product. Whereas a contraceptive pill or food supplement may require daily consumption, other products may be used less frequently.

The average quantity of the product that is used per consumer per use day (F) can be measured in two ways:

f1. Average quantity of the product used per consumer per use day (measured directly via data collected from consumers)

f2. Recommended use or standard portion size

The average quantity of the product used as a direct measure (f1) is a preferred model input, if recent data are available, since it is the more accurate measure. For example, fortified complementary food may be recommended for consumption at 40g per child per day, but it is more accurate to know what is actually consumed by children within the defined area. When feasible, it is usually preferable to disaggregate the average quantity of the product used by consumer subgroups if there are different consumption patterns by subgroup. For example, if children 6-12 months are likely to use more of the product than children 13-24 months, then one could estimate the average quantity of product used by both age groups separately and include both as inputs into the model. Disaggregation could be based on predefined consumer characteristics (eg, age group, gender) in the consuming population or on observed patterns of use (eg, frequent and sporadic users).

The use frequency (G) adjusts for individuals using the product at different frequencies. Some individuals may use soap daily whereas others may use soap once and then discontinue its use. The frequency at which individuals are using the product can be measured in two ways:

g1. Average number of days the consumer uses the product in a defined period (measured directly via data collected from consumers)

g2. Recommended use frequency

The preferable measure is g1, since it is grounded in data collected directly from consumers. When possible, it is preferable to disaggregate use frequency by consumer subgroups if different patterns of utilization by subgroup exist or are of interest to the project. In the absence of detailed use frequency data about subgroups, use frequency (E or G) can be measured as an average across all consumers of the product that could range between occasional and continuous use patterns.

Since razor-and-blade products are a combination of durable goods and fast-moving goods, consumer use can be estimated for the razor using the durable goods model (F2) and/or for the blades using the fast-moving consumer goods model (F3).

## Considerations in using the model

The first step to apply this model to a given intervention is to thoroughly assess which data elements are available.

The model provides a skeleton or structure for a consistent way to estimate the number of beneficiaries from product production, dispatch, sales, or distribution volumes or product coverage data. Once available data sources are identified the model helps to identify all other data elements required to complete the estimate. The model asks to define the target area and population group, time period, and the product quantity used per individual required to interpret and compare results.

When data elements are unavailable or too costly to obtain, then assumptions can be applied. These can be based on similar products (from a super-category or similar categories) in the defined area or on the same product in a similar market outside the defined area.

Where assumptions are necessary, the recommended approach is to identify the extent of variation by defining the best and worst-case scenarios and the resulting product reach estimate variations. For example, if data about the consumer use patterns (F and G) of a fortified staple food product are unavailable, one could assume that individuals consume it at the recommended or a desired quantity and frequency that can achieve an impact (eg, 1 serving x 365 days for a 1-year period). This results in a conservative (low) estimate for product reach. The number of individuals reached would increase if the individual quantity used/consumed is reduced. To see how the product reach estimate changes, the assumption can be applied that all individuals try the product once and then discontinue its use; they consume an average of 1 serving x 1 day per year for a 1-year period. The true consumption frequency is somewhere between these two scenarios, but this would show the range of product reach estimate, or number of individuals the project could be touching based on best knowledge.

If multiple data sources are available, the quality and completeness of each source should be considered. When feasible, multiple measures for the same data element can be calculated and the results triangulated to cross-calibrate validity.

Reach can be estimated for different time periods, eg, for the duration of a 3-year project. All sales can be aggregated for the entire 3-year window to calculate total product reach of the project, or reach can be calculated for each month during the project to show trends. However, monthly product reach should not be added to determine the total project reach as this would result in double-counting individuals who use the product for several months.

Having outlined the model and considerations for applying it, we illustrate its application for a fortified rice project in Brazil. Although Brazil in general cannot be considered a data-scarce setting, most retailers are highly protective of their sales data, which in this case made direct fortified rice consumer sales data unavailable to the project team.

## Brazil case study

The aim of the rice fortification project implemented by PATH and the Global Alliance for Improved Nutrition (GAIN) was to build and test a replicable commercial model to scale up production and distribution of fortified rice through the private sector in Brazil to address micronutrient deficiencies (*14*). While some food fortification programs are mandatory and designed to cover entire populations (*15*), in this case it was voluntary for commercial mills to adopt the fortification technology and for retailers to distribute fortified rice. Therefore, consumers had fortified and unfortified rice options on the shelf and could make dynamic decisions to purchase the fortified product. Product reach, or in this case the number of individuals who were reached with fortified rice, was a key metric that PATH and GAIN were accountable to the donor to measure.

The project area included all regions of Brazil where the fortified rice product was made available by the project’s commercial rice miller partner. The defined time period of interest began in February 2013 when the product was first introduced in stores and available to consumers, until April 2015 when the last supply data was shared with the project.

**Figure 2:**
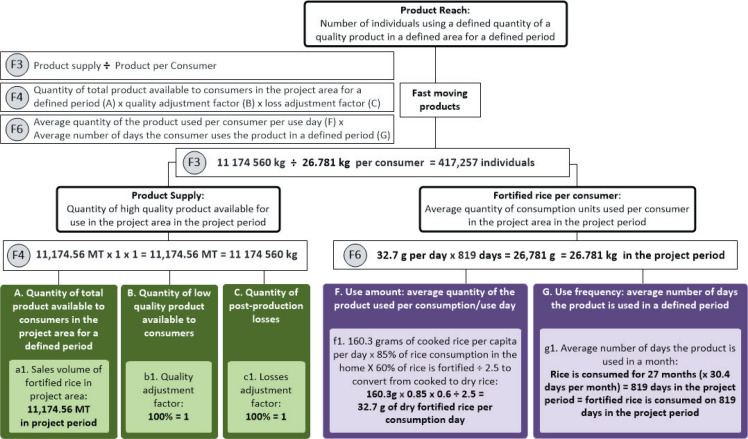
Example of using a model to estimate product reach for a fast moving product.

We applied the fast-moving model and determined the available data sources. We did not have information about the product coverage (H) as no comprehensive coverage surveys had been conducted, and therefore based the reach estimation on product supply and consumption per consumer (**Figure 2**).

We had supply information from the rice miller partner on the total sales per month of fortified rice to retailers by region (a1).

Several rounds of independent testing for micronutrient content, microbiological levels, and organoleptic properties were conducted and found in accordance with Brazilian regulations on fortified foods before the product was introduced in the market. We were thus confident that there were sufficient controls in place to avoid any loss due to supply of low quality product to consumers (B). Hence, we used a quality adjustment factor (b1) of 1. The product dispatched to retailers was expected to be entirely sold by retailers and used by the households in that region, with negligible post-production losses (C), so without any evidence to suggest otherwise, we used a losses adjustment factor (c1) of 1.

Fortified rice is a fast-moving good. A review of existing data was conducted to inform the consumer demand side of the model. From secondary data, we determined that Brazilians consume on average 160.3 grams of cooked rice per capita per day (f1) (*16*). Given that rice is a staple food in Brazil, we assumed that per capita consumption equates to consumption per consumer (which assumes 100% of individuals consume rice in similar quantities). Although this is data for rice in general, we expect that individuals would consume fortified rice in the same quantities as non-fortified rice since they will replace regular rice. The consumed fortified rice amount was adjusted by the fact that 85% of rice consumption is in the home (*16*), and we expect that consumers would only have control over the type of rice used in their home and rice consumed outside of the home would not be fortified. Further, we conducted consumer surveys where individuals were interviewed in retail stores about the frequency of their fortified rice purchases and consumption patterns. This survey found that among those who purchase fortified rice, approximately 60% of their home rice consumption is fortified rice, so we further adjusted the average quantity of the product used by that amount. To normalize this data with sales data, the amount was converted from cooked rice to dry rice by dividing by 2.5, yielding an average amount of fortified rice consumed per individual per day of 32.7 grams. Alternatively, we could have used the daily standard serving size of rice or the recommended serving size based on the amount required to achieve the recommended dietary allowance for iron, zinc, thiamine, and folic acid (f2). We use the average daily consumption (f1) because it is a more accurate measure of how much individuals actually consume.

Rice is a staple food in Brazil and daily consumption data for regular rice is available (*16*). Since we assume fortified rice is consumed as a replacement food, we include daily consumption in the model (g1).

Since the defined period is February 2013 to April 2015 (27 months), g1 = 819 days, then the average quantity of fortified rice consumed per consumption day (f1; 32.7g) is multiplied by the number of days of consumption days (g1; 819 days) to estimate 26.781kg of fortified rice consumed per person in this period (F6). The product supply in the period (F4; 11,174.56MT) is divided by the 26.781kg consumed per person in the period (F6) to calculate product reach in the period (F3; 417,257 people).

We conducted consumer surveys and interviewed individuals in retail stores about the frequency of their fortified rice purchases and consumption patterns. The results revealed that two consumer categories with very different consumption patterns can be distinguished. Consumers that repeatedly purchase fortified rice and others that just try fortified rice for a short period. Two consumer surveys 6 months apart showed similar results, and we applied their average for the project period. Eighty-two percent of fortified rice buyers purchased it repeatedly, and we therefore assumed that only 82% are the same consumers over the period (F3; 417,257 × 0.82 = 342,151 people), they consume 82% of the supply (F4; 11,174.56MT × 0.82 = 9,163.14MT).

The other 18% of the product supply (F4; 11,174.56 – 9,163.14MT = 2011.42MT) are consumed by fortified rice consumers for short consumption periods. We assumed that these consumers consume about 1kg (994g) of fortified rice or 32.7g of fortified rice for one month (30.4 days) on average during the project period.

We estimated that 342,151 repeat consumers and 2,023,561 short term consumers were reached with fortified rice, a total of 2,365,712 individuals in the project period.

In addition to the overall product reach, there were three key subpopulations of interest to the project: 1) women of reproductive age (15-49 years), 2) children 6 months – 5 years old, and 3) individuals from the lower socioeconomic classes (C, D, and E in Brazil’s class structure). To estimate the product reach within these subpopulations, and without any data that would suggest otherwise, we assumed that staple food consumption patterns did not differ significantly across subpopulations. We used existing population data to estimate what fraction of the Brazilian population each target subpopulation represented and multiplied this by the total product reach. As women of reproductive age made up 28.14% of the Brazilian population, children 6 months – 5 years old 6.54% and the lower socioeconomic classes 74.1% of the Brazilian population (*16*), we estimated a product reach of 665,711 women, 154,718 children and 1,752,993 people from the lower socioeconomic classes, respectively.

## DISCUSSION

In this paper, we present the formulation and application of a product reach model that can be equally applied across two categories of goods – durable and fast-moving – and across different intervention or program types that include but are not restricted to immunization, nutrition, reproductive health. There is a paucity in the academic literature of similar models that do not depend on survey data.

As social impact interventions grow in complexity, often involving goods of different types made available to a population, a common approach can be a helpful tool for program managers, funders and decision makers of overarching programs and strategies to:

- estimate reach of the intervention based on different data sources informing product supply and expected consumption patterns,
- provide a common approach to outline the parameters considered in the estimation, and
- achieve consistency in estimations over time, across products, organizations and programs.

While reach does not equate to impact, its value as an intermediate indicator should not be underestimated. The number of individuals using a product with a known positive benefit for the users, such as health products (deworming pills, vaccines, fortified foods or supplements, etc.) is a particularly relevant project endpoint when it is too complex or costly to measure impact indicators or when the impact cannot be easily improved by any one intervention and direct attribution to a particular intervention is less than certain.

Two examples of such hard-to-improve and hard-to-attribute indicators from the world of nutrition are stunting and anemia. It is well-known that both these conditions are multi-factorial, and it is difficult to establish a direct causal link between any particular nutritional or health intervention – for example, consumption of certain foods or use of iron supplements or deworming pills – and improvements in the corresponding indicators (*17*). Programs based on a single intervention to address either of these two issues often show inconsistent results, even though such interventions are known to be beneficial to individuals (*18*). In such situations, reach offers a measure to program evaluation that focuses on actionable and attributable measurement of delivery performance by showing exposure to products.

The model presented herein is sufficiently flexible to address a broad range of goods, settings, contexts, and interventions in a consistent manner. This model provides a standardized approach that can adapt to available data types. It identifies but also enables filling data gaps with assumptions to provide meaningful, timely and actionable data at a reasonable cost. This makes the model particularly useful in contexts where resources for primary data collection are limited by encouraging the use of any available indirect data sources to reduce the information gap. Instead of not being able to review program performance at the beneficiary level when coverage of social impact products is not assessed through surveys, the model calls for using product supply data and measured or estimated product administration or use patterns to estimate current intervention reach and coverage. As with any other model, its output will only be as good as the input data available. While aiming to use the most robust data available, data gaps can be filled with assumptions in the model but need to be adequately explained and reported to consider them when interpreting the results.

Programs usually aim to reach a large number of the target population with an amount of product that will have the desired impact. The metric itself could create incentives counter to the impact goal, because assuming a product use below the amount required to achieve impact translates into higher overall reach estimates in terms of the number of individuals. The model requires a clear definition of the product amount the individual is reached with, which is key when aiming to predict impact. For social impact products this corresponds to the quality, quantity and frequency that would achieve the expected benefit, such as the deworming or vaccination dose frequency required to minimize the risk and consequences of infection.

The model lays out a consistent approach to estimating reach. Reach as the common unit enables the comparison of results from different sources. Frequent reports on product supply may be available to estimate number of people who potentially use a defined quantity of product, while direct consumer/beneficiary data that confirms if the product is actually used in the defined amount by the target population may only be available occasionally. Reach estimates based on product availability or accessibility will keep the focus on routine monitoring data that managers require to manage product delivery and may trigger investments in surveys when appropriate to validate product use at the individual level. When these investments to confirm estimated outcomes are made, the review of validity will also improve assumptions and thereby future estimates made based on more frequently available product supply data. For example, household survey results can also provide further data on selection criteria or decision making of different target groups regarding the product that can be considered in the model for future estimations.

Moreover, a focus on routine monitoring data is cost-effective and will maximize the utility of any investments in additional primary data collection so that they are timed to align with program implementation.

Investments in household surveys that measure low intervention coverage are often not based on data-driven decisions, and would not have been made if monitoring data had been available and used to review maturity of program delivery and expected results (*19*). For example, after a significant break in the supply of deworming tablets or fortified foods the expected number of people having received the product would be low making it an unsuitable moment to invest in a survey to assess coverage. Relying on routine monitoring data in place of primary data collection that requires additional investments will free up resources to improve the program implementation (procurement, distribution and administration of products and services) until expected outcomes in the population require validation through primary data collection.

Reach is a good complementary indicator to coverage that can illustrate magnitude or scale of an initiative particularly when comparing interventions with dissimilar size of target population, but the performance of an intervention in reaching the target population cannot be based on the reach figure alone without relating it to the target population. The model shows the relationship of reach and coverage and how reach estimates based on data from different levels of the product supply chain can be converted to coverage and allows comparison to different coverage measurements for different degrees of interaction between the product and the people (availability, accessibility, acceptability, contact, effectiveness) as described elsewhere for health services (*11*).

Investments into surveys and impact evaluation should only be conducted when product and service delivery monitoring data indicate that outcome and impact are to be expected. The model emphasizes the use of routine monitoring data and provides an approach to how it can be converted to enable regular review of program scale and coverage to identify when it is high and consistent enough to confirm outcomes through surveys and evaluation. Using delivery monitoring data to estimate program reach and coverage will improve data-driven program management: on the one hand ensuring the focus on routine monitoring and reducing premature investments into population surveys to measure actual coverage, while on the other hand keeping a constant eye on progress towards program outcomes and impact.

## CONCLUSIONS

Product reach is a useful metric for program decision-makers when planning and reviewing social impact interventions. This model can be used to estimate product reach based on data from different nodes in the product supply chain, thereby enabling triangulation of results from routine monitoring and coverage survey data. It generates results that can show policymakers how their investments benefit the target population even if direct population level data are not available and provides a basis to expand on with more robust outcome and impact measures for program evaluation. The model facilitates data-driven decision-making for managers of social impact interventions and the use of routine monitoring to ensure progress towards program outcomes and impact.

The model can be applied to a wide range of durable and fast moving social impact products. An interesting extension to the model would be to apply it to interventions that provide services and product-service bundles. We encourage others to apply the model and share their specific use cases, key data sources used, and any necessary adaptations made to increase its robustness or to demonstrate its applicability to a wide range of social impact interventions.
